# Tocolysis and Neurodevelopment of Children Born Very Preterm

**DOI:** 10.1001/jamanetworkopen.2024.42602

**Published:** 2024-10-31

**Authors:** Thibault Plouchart, Thibaut Sabatier, Jean-Baptiste Muller, Gaëlle Pinto Cardoso, Loïc Sentilhes, Jacques Bénichou, Stéphane Marret

**Affiliations:** 1 Department of Neonatal Pediatrics, Intensive Care and Neuropediatrics, Rouen University Hospital, Rouen, France; 2 Paediatric Intensive Care Unit, Caen University Hospital, Rouen University Hospital, Caen, France; 3 Department of Epidemiology and Health Promotion, Rouen University Hospital, Rouen, France; 4 Department of Obstetrics and Gynecology, Bordeaux University Hospital, Bordeaux, France; 5 Department of Biostatistics, Rouen University Hospital, Rouen, France; 6 L'Institut National de la Santé et de la Recherche Médicale U1018, Centre de Recherche en Epidémiologie et Santé des Populations, Paris-Saclay University, Paris, France; 7 L'Institut National de la Santé et de la Recherche Médicale U1245, Normandy University, France

## Abstract

**Importance:**

Neurodevelopmental outcomes of very preterm children exposed to tocolytics are not well described.

**Objective:**

To investigate whether tocolysis administered after spontaneous preterm labor is associated with neurodevelopmental outcomes at 5.5 years and to assess whether the type of tocolytic drug is associated with neurodevelopmental outcomes among infants exposed.

**Design, Setting, and Participants:**

This prospective, national, population-based cohort study used data from the French Etude Épidémiologique sur les Petits Âges Gestationnels–2 cohort. Children who were alive and participated in an assessment at 5.5 years and whose mothers experienced spontaneous preterm labor without an infectious context and delivered at 24 to 31 weeks were eligible for this study. Recruitment occurred from March to December 2011. Follow-up at age 5.5 years was conducted from September 2016 to December 2017. Data analysis was performed from July 2023 through April 2024.

**Exposures:**

The primary analysis examined tocolytics (yes vs no), and the secondary analysis examined the type of tocolytic (atosiban vs calcium channel blockers [CCBs]).

**Main Outcome and Measure:**

The composite outcome neurodevelopmental disabilities included cerebral palsy; visual, hearing, and cognitive deficiencies; developmental coordination disorders; or behavioral problems.

**Results:**

A total of 1055 mothers (mean [SD] age, 29.2 [5.7] years) had preterm labor without fever and gave birth to 1320 children (704 male [weighted percentage, 53.3%; 95% CI, 50.6%-56.1%]; mean [SD] gestational age, 28.8 [2.0] weeks). Overall, 776 mothers (weighted percentage, 73.5%; 95% CI, 70.8%-76.2%) received tocolytics; 136 mothers (weighted percentage, 17.9%; 95% CI, 15.3%-20.8%) received only a CCB, and 295 mothers (weighted percentage, 37.6%; 95% CI, 34.2%-41.0%) received only atosiban. From modified Poisson regression with propensity score matching, the risk of overall neurodevelopmental disabilities (mild, moderate, or severe) at 5.5 years did not differ between preterm children exposed and not exposed to tocolytics (relative risk [RR], 1.11; 95% CI, 0.85-1.45; *P* = .44) or in preterm infants exposed to atosiban compared with those exposed to CCBs (RR, 0.94; 95% CI, 0.67-1.32; *P* = .71).

**Conclusions and Relevance:**

In this study, tocolytics were not associated with neurodevelopmental disabilities among very preterm children surviving at 5.5 years.

## Key Points

**Question:**

Is tocolysis associated with an increased risk of neurodevelopmental disabilities in very preterm infants at 5.5 years?

**Findings:**

In this cohort study of 1055 mothers of 1320 children who experienced preterm labor and delivered at 24 to 31 weeks of gestation, the risk of overall neurodevelopmental disabilities at 5.5 years did not significantly differ with vs without antenatal exposure to tocolysis. Among infants exposed, there were no differences in outcomes associated with atosiban vs calcium channel blocker exposure.

**Meaning:**

In this study, in utero tocolytic exposure was not associated with increased or decreased risk of neurodevelopmental disabilities during early childhood.

## Teaser

This cohort study investigates the association of tocolysis with risk of neurodevelopmental disabilities at 5.5 years among infants born very preterm.

## Introduction

Prematurity, defined as birth before 37 weeks of gestation, is a global public health burden due to its high prevalence, with 15 million premature births per year accounting for 11% of all births worldwide,^1,2^ and its consequences for morbidity and mortality. Preterm birth is the main cause of mortality in children younger than 5 years.^3^ Children born preterm may also face a higher risk of developmental challenges, including cerebral palsy, cognitive impairment, deafness, blindness, and autism spectrum disorders, as well as more subtle impairment, such as developmental coordination disorders, neurovisual dysfunctions, specific language and learning disorders, executive function impairment, attention-deficit/hyperactivity disorder, or behavioral problems.^4,5^ Overall, approximately 30% to 35% of very preterm children have mild disabilities by age 5.5 years.^6,7,8^ These findings emphasize the strong need to reduce premature birth incidence to prevent its detrimental consequences.

Tocolytics are used to prevent preterm birth through the induction of smooth muscle relaxation, which helps suppress uterine contractions.^9^ In delaying birth, these medications provide an opportunity for antenatal optimization. This entails administering corticosteroids for fetal lung maturation, magnesium sulfate for neuroprotection, and antibiotics for group B *Streptococcus* prevention and allowing time for the expectant individual to be transferred to a facility equipped with adequate neonatal care resources. This therapeutic approach is endorsed by international academic societies for its efficacity in managing cases of preterm labor.^10^ In France, the 2 main tocolytics drug classes used are calcium channel blockers (CCBs), such as nifedipine or nicardipine, and oxytocin receptor antagonists, such as atosiban.^11^ The safety of tocolysis for the fetal brain remains uncertain owing to insufficient data. Nifedipine has been associated with a redistribution of the fetal lamb circulation when administered to ewe,^12^ and fetal death has been reported in humans,^13^ albeit as a rare adverse effect. In contrast, nifedipine may provide some protection from glutamate toxicity in cortical neurons.^14^ Atosiban, as an antagonist of oxytocin, could prevent the release of oxytocin, which favors mother-infant interactions after birth and may compensate for potential deleterious effects of stress.^15^ Using data from the Etude Épidémiologique sur les Petits Âges Gestationnels–2 (EPIPAGE-2) cohort study, our team previously reported that the use of tocolytics was associated with reduced risk of neonatal death and severe intraventricular hemorrhage before hospital discharge for preterm infants born between 24 and 31 weeks of gestation after spontaneous labor.^16^ A 2022 meta-analysis^11^ of 122 trials concluded that the association between tocolytic use and neonatal outcomes was uncertain. Furthermore, few studies have evaluated the association between antenatal exposure to tocolytics and long-term neurodevelopment^17,18,19,20^^,^

We aimed to investigate whether tocolysis administered after spontaneous preterm labor was associated with neurodevelopmental disabilities at 5.5 years in children born very preterm overall. Additionally, we aimed to assess whether the tocolytic type (atosiban vs CCBs) was associated with neurodevelopmental disabilities among infants exposed.

## Methods

### Data Collection

This cohort study is reported following the Strengthening the Reporting of Observational Studies in Epidemiology (STROBE) reporting guideline for cohort analyses. EPIPAGE-2 is a prospective, national, population-based cohort of children born preterm (ie, at 22-34 weeks of gestation) in 2011 in 25 French regions (all French regions except 1).^21^ Recruitment was conducted over an 8-month period for births at 22 to 26 weeks’ gestation and a 6-month period for births at 27 to 31 weeks’ gestation. Among the 8400 children eligible at birth, 7804 newborns were included at baseline (participation rate: 93.0%). Of those, 5170 children were liveborn with parental consent. A comprehensive neurodevelopmental assessment was proposed to families of all 4441 children surviving at age 5.5 years.^6^ Parents completed a self-administrated questionnaire, and children had a clinical examination by a physician and a cognitive assessment by a neuropsychologist that were performed in dedicated examination centers. All professionals were trained to ensure homogeneity in data collection. At infant age 5.5 years, at least 1 assessment was performed for 3083 children (69.4%). Full details of the cohort recruitment and data collection were reported elsewhere.^21,22^

The EPIPAGE-2 study was approved by the National Data Protection Authority and by appropriate ethics committees (the Consultative Committee on the Treatment of Data on Personal Health for Research Purposes and Committee for the Protection of People Participating in Biomedical Research). Assessments occurred only after families had received information and provided written informed consent. The EPIPAGE-2 study’s institutional review board approvals and satisfaction of informed consent requirements extended to this study.

### Study Population

In this study, we included children whose mothers experienced spontaneous preterm labor (including preterm, prelabor rupture of membranes or with intact membranes) and delivered at gestational ages 24 to 31 weeks, with children surviving up to age 5.5 years. We excluded 1328 children with terminations of pregnancies, 755 children with fetal death before maternal admission to the hospital, and 137 children with lethal malformations. Furthermore, we excluded children whose mothers were experiencing an infectious context (ie, mothers with infectious complications, body temperature ≥38.5 °C during delivery, or clinical chorioamnionitis) because tocolysis is contraindicated in this context; we also excluded children whose mothers had pregnancy-related vascular disease (hypertension, preeclampsia or eclampsia, placental abruption, or hemolysis, elevated liver enzymes, and low platelets) because there is no indication for tocolysis in cases of pregnancy-related vascular disease. Children born with fetal growth restriction were also excluded. Finally, we excluded 22 mothers with missing data on tocolytic treatment exposure (Figure).

**Figure.**
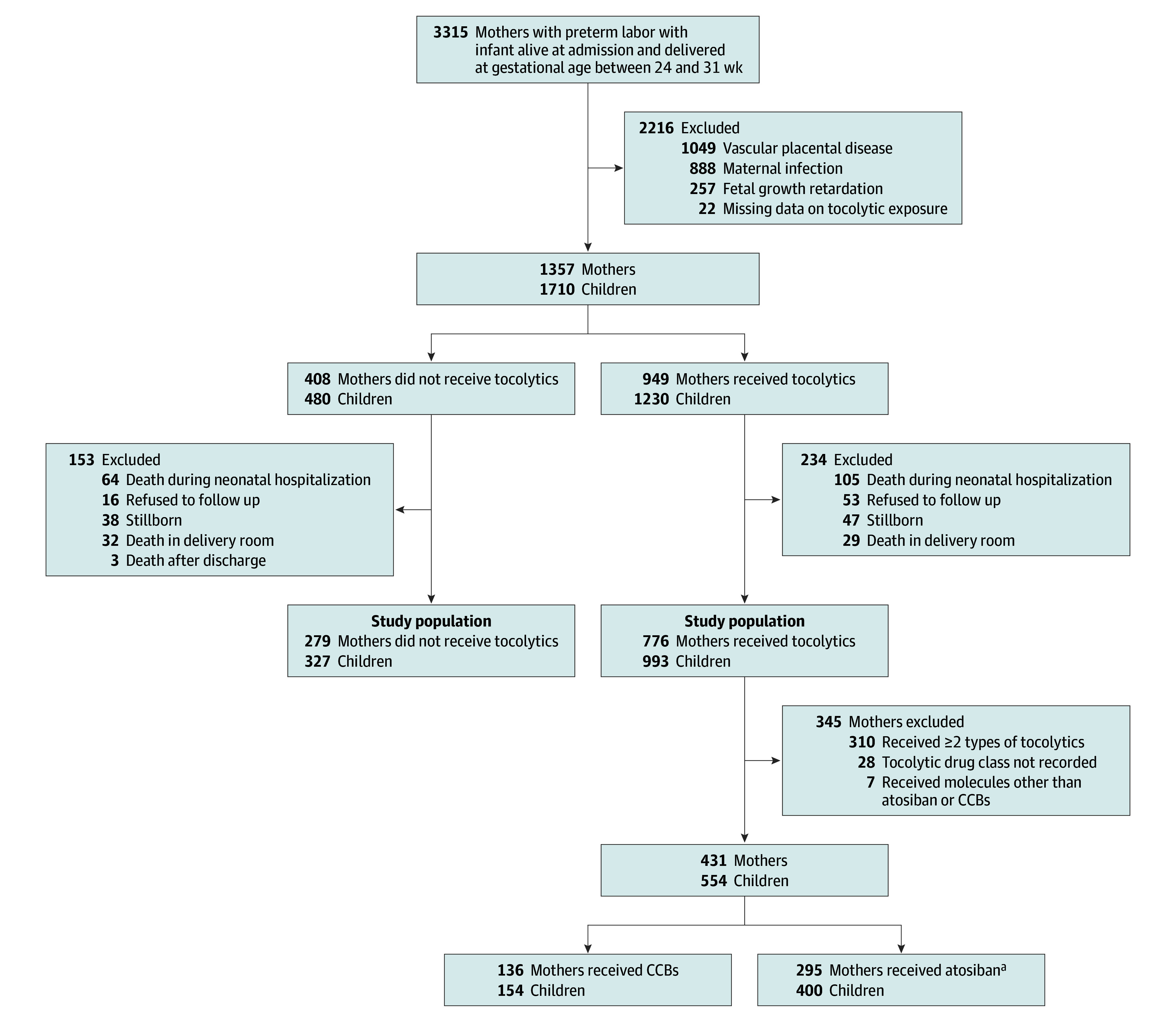
Study Flowchart CCB indicates calcium channel blocker. ^a^No other oxytocin antagonist was used.

### Exposure

For the main analysis on the association between tocolysis exposure and neurodevelopmental disabilities, we considered mothers exposed if they received any tocolytic treatment during their last hospitalization ending in delivery. In the secondary analysis comparing tocolytic treatments (atosiban vs CCBs), the sample used for the analysis consisted of women who received a single tocolytic treatment class (either atosiban or CCB) during the hospitalization that ended with delivery. Hence, this analysis excluded 310 mothers who had sequentially been exposed to several tocolytic classes and 7 mothers exposed to molecules other than atosiban or CCB in their last hospitalization ending in delivery (Figure).

### Outcome Measures

Neurodevelopmental status at age 5.5 years was defined as a composite of cerebral palsy; visual, hearing, or cognitive deficiencies; behavioral problems; or developmental coordination disorders, including mild, moderate, and severe forms.^6^ Mild disabilities were defined as 1 or more of the following: mild cerebral palsy (Gross Motor Function Classification System [GMFCS] score of 1), a visual disability of 3.2 of 10 to less than 5 of 10 (Sander-Zanlonghi scale), unilateral or bilateral hearing loss of less than 40 dB, a Full Scale Intelligence Quotient (FSIQ) score between −2 and −1 SDs from the mean (Wechsler Preschool and Primary Scale of Intelligence, 4th edition), developmental coordination disorders (Movement Assessment Battery for Children, 2nd edition, total score ≤5th percentile), behavioral difficulties (Strengths and Difficulties Questionnaire, total score ≥90th percentile). Moderate neurodevelopmental disabilities included 1 or more of the following: cerebral palsy with a GMFCS score of 2 or 3, a bilateral binocular visual acuity of 1 of 10 to less than 3.2 of 10 (Sander-Zanlonghi scale), unilateral or bilateral hearing loss of 40 to 70 dB, or a FSIQ score −3 to −2 SDs from the mean. Severe neurodevelopmental disabilities were defined as 1 or more of the following: severe cerebral palsy (a GMFCS score of 4 or 5), a bilateral binocular visual acuity less than 1 of 10 (Sander-Zanlonghi scale), unilateral or bilateral hearing loss greater than 70 dB, or a FSIQ score less than −3 SDs from the mean. Neurodevelopmental status was analyzed as a dichotomous outcome: no disabilities vs any disabilities (including mild, moderate or severe disabilities).

### Covariates

We considered the following variables in the analysis: Sociodemographic characteristics consisted of maternal age (<25, 25-34, and ≥35 years), country of origin (France or elsewhere in Europe, North African countries, other African countries, and other country), level of education (<high school, high school or 1-2 years of graduate studies, and ≥3 years of graduate studies), smoking during pregnancy, and parental socioeconomic position (professional, intermediate, administrative or public service, service worker, manual worker, and unemployed), which was defined by the highest occupational position between the occupations of the parents or of the mother only if living alone. Clinical factors consisted of single or multiple pregnancy and gestational age at admission (<27, 27-29, and 30-31 weeks), and obstetrical management consisted of in utero transfer, infertility treatment administration of antenatal corticosteroids (≥1 injection vs no injection) or antenatal magnesium sulfate, and birth in a tertiary care center.

### Statistical Analysis

Maternal and child characteristics were described as frequencies and percentages in each exposure group (with and without tocolysis and CCBs or atosiban). Percentages were weighted according to the duration of the recruitment period (in weeks) for each gestational age group; weights were 1.0 for births at 24 to 26 weeks’ gestation and 1.34 for births at 27 to 31 weeks’ gestation. Weighting allowed us to account for the sampling scheme of the cohort and provide appropriate figures for all births between 24 and 31 weeks of gestation.

We used the same analysis strategies for primary and secondary objectives, using propensity score analysis to reduce indication bias. The propensity score was defined as the participant’s probability of receiving a specific treatment (ie, tocolysis yes vs no or atosiban vs CCBs) conditional on observed baseline characteristics; it was estimated using multivariable logistic regression. Then, 1:1 greedy nearest-neighbor matching was used without replacement within a caliper of 0.1 SD of the logit of the propensity score. We included all previously listed covariates in the propensity score model. Then, modified Poisson regression using generalized estimation equations to account for correlation between twins was applied to exposed vs unexposed pairs (or atosiban vs CCBs pairs) matched on the propensity score for tocolysis exposure to investigate the association between tocolytic exposure and neurodevelopmental status (ie, no disabilities vs any disabilities).^23^ To account for missing data for covariates included in the propensity score (missing proportions from 0% to 5%) and main outcome, which was missing in 509 children (38.6%) overall, multiple imputation was performed by fully conditional specification,^24^ with 20 imputations and 30 iterations each; proportions of missing data regarding the outcome were similar among children exposed (380 of 993 children [38.3%]) and not exposed (129 of 327 children [39.4%]) to tocolytics. Among methods available for combining multiple imputation with the propensity score approach,^25,26,27^ we used the passive approach.^27^ This consists of first imputing covariates used to estimate the propensity score, then estimating the propensity score and fitting a modified Poisson regression for each resulting dataset to obtain estimates of the association between the exposure and main outcome, and finally combining these estimates to produce an overall estimate (the within approach). This approach has been shown to produce unbiased estimates with appropriate CIs.^25^ We performed 3 sensitivity analyses, 1 using the same approach as described previously but without multiple imputation (ie, complete case analysis [model 2]) and 2 applying inverse probability of treatment weighting with the propensity score to the whole sample rather than propensity score matching,^28^ either with multiple imputation (model 3) or complete case analysis (model 4). Relative risks (RRs) and their corresponding 95% CIs were estimated. All analyses were performed with R statistical software version 4.3.1 (R Project for Statistical Computing), and statistical significance was defined as a 2-sided *P* < .05. Data analysis was performed from July 2023 through April 2024

## Results

The sample for primary analyses consisted of 1055 mothers (mean [SD] age, 29.2 [5.7] years) who gave birth to 1320 children (mean [SD] gestational age 28.8 [2.0] weeks; 704 male [weighted percentage, 53.3%; 95% CI, 50.6%-56.1%]). Overall, 776 mothers (weighted percentage, 73.5%; 95% CI, 70.8%-76.2%) received tocolytics. Premature rupture of membranes occurred among 406 of 1046 mothers with data (weighted percentage, 39.3%; 95%CI, 36.3%-42.3%). There were 616 children born via cesarean delivery among 1304 children with data (weighted percentage, 47.7%; 95% CI, 44.9%-50.4%). Baseline sociodemographic and clinical characteristics by exposure to tocolysis are reported in Table 1. The 414 mothers of 509 children who did not participate at the assessment at 5.5 years were younger, had a lower educational level and socioeconomic status, and were more frequently smokers than those who participated. However, the 2 groups did not differ in obstetrical characteristics or treatment, including tocolytic administration, except for infertility treatment and term of pregnancy at mother’s admission (eTable 1 in Supplement 1).

**Table 1.** Baseline Characteristics by Tocolytic Exposure

Characteristic	Mothers, No. (% [95% CI]) (N = 1055)^a^	
	No tocolysis (n = 279)	Tocolysis (n = 776)
Maternal characteristics		
Age, y		
<25	55 (19.3 [4.9-24.2])	146 (18.8 [16.1-21.7])
25-34	170 (61.0 [55.1-66.7])	504 (65.0 [61.5-68.3])
≥35	54 (19.7 [15.3-24.8])	126 (16.2 [13.7-19.0])
Mother’s country of origin (n = 1048)		
France or other European country	217 (78.9 [73.8-83.4])	644 (83.6 [80.8-86.1])
North African country	28 (10.1 [6.9-14.1])	49 (6.5 [4.9-8.4])
Other African country	15 (5.0 [2.9-7.9])	46 (5.8 [4.3-7.5])
Other	17 (6.0 [3.6-9.1])	32 (4.1 [2.9-5.7])
Level of education (n = 997)		
<High school	89 (35.5 [29.6-41.6])	220 (29.7 [26.5-33.1])
High school or 1-2 y of graduate studies	111 (43.3 [37.2-49.6])	319 (42.9 [39.3-46.5])
≥3 y o Graduate studies	52 (21.2 [16.4-26.7])	206 (27.4 [24.3-30.7])
Parents’ socioeconomic position (n = 1009)		
Professional	51 (20.1 [15.4-25.3])	190 (25.1 [22.1-28.3])
Intermediate	35 (13.7 [9.8-18.3])	164 (21.9 [19.0-25.0])
Administrative or public service	81 (31.3 [25.8-37.2])	205 (27.7 [24.5-31.0])
Service worker	36 (13.0 [9.3-17.3])	90 (11.9 [9.7-14.4])
Manual worker	39 (15.0 [11.0-19.7])	81 (10.8 [8.7-13.1])
Unemployed	18 (6.9 [4.2-10.5])	19 (2.6 [1.6-3.9])
Smoking during pregnancy (n = 1021)	65 (24.3 [19.4-29.8])	173 (23.2 [20.3-26.4])
Obstetrical characteristics and management		
Twin gestation	62 (22.4 [17.7-27.6])	244 (31.4 [28.1-34.7])
Infertility treatment (n = 1016)	33 (12.3 [8.7-16.7])	126 (16.4 [13.9-19.2])
Antenatal corticosteroid (n = 1025)	86 (32.4 [26.9-38.2])	521 (69.1 [65.8-72.4])
In utero transfer (n = 1047)	30 (10.8 [7.5-14.8])	433 (56.2 [52.6-59.7])
Antenatal magnesium sulfate (n = 1041)	7 (2.7 [1.1-5.1])	38 (5.0 [3.6-6.7])
Term of pregnancy at mother’s admission, (n = 1050), wk		
<27	65 (19.5 [15.4-24.2])	250 (28.8 [25.7-31.9])
27-29	103 (38.9 [33.2-44.9])	280 (38.2 [34.8-41.7])
30-31	110 (41.6 [35.8-47.5])	242 (33.0 [29.7-36.5])
Gestational age at birth, wk^b^		
24-27	81 (25.5 [20.7-30.6])	219 (24.4 [21.5-27.3])
28-31	198 (74.5 [69.4-79.3])	557 (75.6 [72.7-78.5])
Maternity unit characteristic: birth in tertiary care center	180 (64.8 [59.0-70.3])	656 (84.4 [81.7-86.9])

^a^ Denominators vary by the number of mothers with missing data for each variable. Percentages are weighted to account for varying survey durations among gestational age groups.

^b^ This variable was not included in the propensity score analysis because it may be associated with treatment.

The sample for secondary analysis consisted of 431 mothers (mean [SD] age, 29.0 [5.5] years) who gave birth to 554 children (284 male [weighted percentage, 51.5%; 95% CI, 47.3%-55.7%]), among whom 295 mothers (weighted percentage, 67.7%; 95% CI, 63.1%-72.1%; 37.6% of all mothers; 95% CI, 34.2%-41.0%) received atosiban (no other oxytocin receptor antagonist was used) and 136 mothers received CCBs (weighted percentage, 32.3%; 95% CI, 27.9%-36.9%; 17.9% of all mothers; 95% CI, 15.3%-20.8%). Baseline sociodemographic and clinical characteristics by tocolytic drug class are presented in Table 2. At the 5.5-year follow-up, 306 of 613 children exposed with data on the outcome (weighted percentage, 49.7%; 95% CI, 45.7%-53.7%) and 99 of 198 children not exposed with data on the outcome (weighted percentage, 49.3%; 95% CI, 42.3%-56.4%) had neurodevelopmental disabilities. Among children with neurodevelopmental disabilities and exposed to tocolysis, 213 children (weighted percentage, 70.1%; 95% CI, 64.7 %-75.1%) had mild disabilities, 67 children (weighted percentage, 21.8%; 95% CI, 17.4%-26.7%) had moderate disabilities, and 26 children (weighted percentage, 8.1%; 95% CI, 5.4%-11.5%) had severe disabilities, while corresponding figures were 65 children (weighted percentage, 66.1%; 95% CI, 56.3%-75.2%), 19 children (weighted percentage, 19.5%; 95% CI, 12.3%-28.3%), and 15 children (weighted percentage, 14.4%; 95% CI, 8.4%-22.2%), respectively, in the absence of tocolysis. Additionally, at the 5.5-year follow-up, 125 of 239 children with data on the outcome (weighted percentage, 51.6%; 95% CI, 45.2%-58.0%) and 44 of 91 children with data on the outcome (weighted percentage, 48.0%; 95% CI, 37.7%-58.5%) in atosiban and CCBs groups, respectively, had neurodevelopmental disabilities. Among children with neurodevelopmental disabilities who were exposed to atosiban, 84 children (weighted percentage, 67.6%; 95% CI, 58.9%-75.6%) had mild disabilities, 25 children (weighted percentage, 20.1%; 95% CI, 13.6%-27.9%) had moderate disabilities, and 16 children (weighted percentage, 12.3%; 95% CI, 7.3%-18.9%) had severe disabilities, whereas corresponding figures were 31 children (weighted percentage, 70.6%; 95% CI, 55.5%-83.1%), 11 children (weighted percentage, 25.3%; 95% CI, 13.6%-40.1%), and 2 children (weighted percentage, 4.1%; 95% CI, 0.6%-12.7%), respectively, in case of CCB exposure.

**Table 2.** Baseline Characteristics by Tocolytic Treatment Among Mothers With Single Tocolytic Treatment

Characteristic	Mothers, No. (% [95% CI]) (n = 431)^a^	
	CCB (n = 136)	Atosiban (n = 295)
Maternal characteristics		
Age, y		
<25	25 (18.4 [12.4-25.6])	63 (21.6 [17.1-26.6])
25-34	88 (64.6 [56.2-72.4])	187 (63.3 [57.6-68.7])
≥35	23 (17.0 [11.3-24.1])	45 (15.1 [11.3-19.6])
Mother’s country of origin (n = 427)		
France or other European country	104 (76.7 [69.0 -83.4])	250 (85.9 [81.6-89.6])
North African country	10 (7.6 [3.8-13.0])	15 (5.3 [3.1-8.4])
Other African country	13 (9.3 [5.1-14.9])	15 (5.0 [2.9 -7.9])
Other	9 (6.4 [3.1-11.4])	11 (3.8 [1.9-6.4])
Level of education (n = 415)		
<High school	43 (32.5 [24.7-40.9])	94 (33.4 [28.0 -39.1])
High school or 1-2 y of graduate studies	54 (41.1 [32.8-49.8])	115 (40.4 [34.7-46.2])
≥3 y Graduate studies	34 (26.4 [19.2-34.5])	75 (26.2 [21.2-31.6])
Parents’ socioeconomic position (n = 415)		
Professional	33 (25.4 [18.4-33.4])	54 (18.5 [14.3-23.4])
Intermediate	26 (19.3 [13.1-26.7])	72 (25.7 [20.6-30.9])
Administrative or public service	35 (26.6 [19.4-34.7])	84 (30.6 [25.3-36.2])
Service worker	17 (12.9 [7.8-19.4])	33 (11.3 [8.0-15.4])
Manual worker	13 (10.0 [5.6-16.0])	33 (11.4 [8.0-15.5])
Unemployed	8 (5.8 [2.7-10.7])	7 (2.5 [1.1-4.9])
Smoking during pregnancy (n = 419)	32 (23.9 [17.1-31.7])	70 (24.9 [20.1-30.3])
Obstetrical characteristics and management		
Twin gestation	19 (13.8 [8.7-20.4])	123 (42.2 [36.5-47.9])
Infertility treatment (n = 416)	14 (10.3 [5.9-16.3])	49 (17.0 [12.9-21.7])
Antenatal corticosteroid (n = 423)	91 (68.0 [59.6-75.7])	162 (56.4 [50.6-62.1])
In utero transfer (n = 428)	54 (39.6 [31.5-48.1])	175 (60.3 [54.5-65.8])
Antenatal magnesium sulfate (n = 422)	4 (3.1 [0.9-7.0])	17 (5.8 [3.5-9.0])
Term of pregnancy at mother’s admission (n = 429), wk		
<27	21 (12.8 [8.1-18.7])	93 (27.2 [22.5-32.3])
27-29	49 (37.2 [29.2-45.6])	111 (40.4 [34.8-46.2])
30-31	66 (50.0 [41.6-58.5])	89 (32.4 [27.1-38.0])
Gestational age at birth, wk^b^		
24-27	26 (16.6 [11.1-23.2])	96 (28.0 [23.2-33.2])
28-31	110 (83.4 [76.8-88.8])	199 (72.0 [66.8-76.8])
Maternity unit characteristic: birth in a tertiary care center	110 (80.7 [73.3-86.8])	232 (78.3 [73.3-82.9])

Abbreviation: CCB, calcium channel blocker.

^a^ Denominators vary by the number of mothers with missing data for each variable. Percentages are weighted to account for varying survey durations among gestational age groups.

^b^ This variable was not included in the propensity score analysis because it may be associated with treatment.

After propensity score matching, maternal and pregnancy characteristics were well balanced overall according to tocolytic exposure (yes vs no) (eFigure 1 in Supplement 1) and according to exposure to CCBs or atosiban in case of tocolysis (eFigure 2 in Supplement 1). The risk of overall neurodevelopmental disabilities at 5.5 years was not significantly different between preterm infants exposed and not exposed to tocolytics in the main model (RR for tocolytic exposure, 1.11; 95% CI, 0.85-1.45; *P* = .44) (Table 3). Sensitivity analyses on complete cases or using inverse probability weighting yielded very close estimates of RR, still with no association between tocolytic exposure and overall risk of neurodevelopmental disabilities (Table 3).

**Table 3.** Association Between Tocolytic Exposure and Neurodevelopmental Status at Age 5.5 y

Exposure	Neurodevelopmental disability, aRR (95% CI)^a^			
	Main model^b^	Model 2^c^	Model 3^d^	Model 4^e^
Tocolytic exposure				
Children, No.	546	232	1320	697
No	1 [Reference]	1 [Reference]	1 [Reference]	1 [Reference]
Yes	1.11 (0.85-1.45)	1.12 (0.85-1.46)	1.07 (0.81-1.41)	1.07 (0.72-1.60)
Type of tocolytic exposure				
Children, No.	240	98	554	296
CCB	1 [Reference]	1 [Reference]	1 [Reference]	1 [Reference]
Atosiban	0.94 (0.67-1.32)	1.16 (0.81-1.66)	0.91 (0.66-1.26)	1.00 (0.65-1.52)

Abbreviations: aRR, adjusted relative risk; CCB, calcium channel blocker.

^a^ All models are modified Poisson regression models and accounted for maternal age, geographic origin of the mother, level of education, household sociodemographic position, smoking during pregnancy, level of neonatal intensive care of the maternity unit, infertility treatment, single or multiple pregnancy, antenatal magnesium sulfate use, antenatal corticosteroid use, in utero transfer, and gestational age at admission using propensity scores.

^b^ Model 1 used 1:1 propensity score matching after multiple imputation.

^c^ Model 2 used 1:1 propensity score matching in complete case analysis.

^d^ Model 3 used inverse probability of treatment weighting after multiple imputation.

^e^ Model 4 used inverse probability of treatment weighting in the complete case analysis.

The risk of overall neurodevelopmental disabilities was not significantly different between preterm infants exposed to CCBs and those exposed to atosiban (RR for atosiban vs CCBs, 0.94; 95% CI, 0.67-1.32; *P* = .71) (Table 3). Sensitivity analyses gave comparable results (Table 3).

## Discussion

In this cohort study, we found no association between exposure to tocolytics and the risk of neurodevelopmental disabilities at age 5.5 years. Moreover, in case of in utero exposure to tocolytics, no significant difference in the risk of neurodevelopmental disabilities was found between infants exposed to CCBs and those exposed to atosiban.

In a previous study based on EPIPAGE-2 data,^16^ authors found that tocolysis was associated with a benefit for neonatal outcomes (ie, neonatal death, severe intraventricular hemorrhage [grade III-IV according to Papile classification^29^]) at hospital discharge. However, these short-term benefits did not translate into long-term benefits in our study.

Our study results are concordant with those of the Assessment of Perinatal Outcome after Specific Tocolysis in Early Labour III (APOSTEL III) study,^19^ a follow-up study of an open label multicenter randomized clinical trial. APOSTEL III did not find a significant difference between nifedipine and atosiban in neurodevelopmental outcomes of children whose mothers had preterm labor and received tocolysis with nifedipine or atosiban. In that study, the main outcome was a composite of abnormal neurodevelopment assessed between ages 2.5 and 5.5 years. Development was considered abnormal overall if the child scored as abnormal on at least 1 the following parental questionnaires: the Ages and Stages Questionnaire, Behavioral Rating Inventory of Executive Function, and Child Behavior Checklist. Our study relied on a much more accurate 5-year assessment with reference tools (ie, including intellectual quotient and developmental coordination disorders).

The combined results of our study and those of the APOSTEL III study are reassuring with respect to the potential adverse neurodevelopmental outcomes during childhood after use of oxytocin receptor antagonists. Nevertheless, these results should not overshadow pharmacological data on the effect of oxytocin. Indeed, oxytocin plays a central role in establishing the mother-child bond and the child behavioral profile.^30^ The use of synthetic oxytocin to facilitate labor and delivery has been reported to be associated with a higher risk of autistic spectrum disorder for the child,^31,32^ and conversely, a low oxytocin blood level was shown to be associated with an increased risk of autistic spectrum disorder in a 2021 meta-analysis.^33^ Moreover, the main CCB used as a tocolytic treatment is nifedipine, which has been shown to produce deleterious effects on mother and fetal hemodynamics in an animal model using sheep,^34^ although it was shown more recently that these hemodynamic adverse effects were uncertain in human fetuses,^35^ which was probably associated with daily doses.^36^

Neurodevelopmental disabilities in early childhood are commonly reported as a composite outcome of cerebral palsy and sensory and cognitive impairment^7,8^ (ie, severe or moderate neurodevelopmental disabilities). Behavioral difficulties and developmental coordination disorders (ie, mild neurodevelopmental disabilities^6^) represent more subtle disorders that are usually reported separately.^37,38^ Nevertheless, they may alter a child’s socioemotional competencies substantially. Moreover, they are often associated with more severe disorders. Therefore, we included them in the composite of neurodevelopmental disabilities (mild, moderate, or severe) to more fully account for the complexity of challenges faced by children born very preterm. However, differential associations of tocolytic treatments with separate neurodevelopmental disorders cannot be ruled out, and further research based on larger studies could be useful to better understand which specific neurocognitive processes may be disrupted by tocolytics.

Strengths of this study include the longitudinal and population-based design of the EPIPAGE-2 cohort study and almost exhaustive nationwide recruitment of mothers with preterm labor and their children. Moreover, the assessment of neurodevelopment disorders at 5.5 years was standardized and performed without knowledge of tocolytic exposure before delivery. Additionally, several sensitivity analyses were performed and yielded results consistent with the main analysis, suggesting the robustness of our findings.

### Limitations

The 4 main limitations of this study are related to its longitudinal and observational design. Participant attrition, inherent to long-term follow-up studies, resulted in a missing outcome in 509 children (38.6%); however, proportions were similar among children exposed to tocolytics (380 of 993 children [38.3%]) and those not exposed (129 of 327 children [39.4%]) to tocolytics. It is reassuring in this respect that analyses with multiple imputation and complete cases analyses yielded similar results. Furthermore, because the study was observational, the administration of tocolytics was not random and may have been determined by maternal or pregnancy characteristics. To control for this potential indication bias, we conducted a propensity score analysis. However, residual confounding remains possible because potentially important information, such as the position of obstetric teams with regard to the treatment of very preterm infants, was not recorded in the EPIPAGE-2 cohort. Third, given that this study was restricted to very preterm births, gestational age at birth may have acted as a collider if it lay on the pathway between (tocolytic) exposure and (neurodevelopmental) outcomes. This would result in stratification-collider bias^39^ and may have yielded attenuation of the association between tocolytic exposure and neurodevelopmental disability outcomes in our analysis. Randomization appears to be the only unequivocal solution to control for this bias in this context. Of note, a randomized clinical trial on tocolysis in women experiencing preterm prelabor rupture of membranes is currently underway.^40^ Fourth, although the EPIPAGE-2 cohort study was large and included 4441 children who were born before 35 weeks of gestational age and had survived to age 5.5 years, statistical power was limited for associations of small magnitude like those found in these analyses. Indeed, statistical power was less than 50% for RR values of 1.30 or less for tocolytic exposure with respect to the main model, so associations of small magnitude cannot be ruled out. On the contrary, statistical power was greater than 95% for RR values of 1.70 or greater, so large associations of tocolytic exposure with neurodevelopmental risk can be ruled out safely. Similarly, for comparisons of CCBs and atosiban, statistical power was less than 50% for RR values of 0.72 or more (atosiban relative to CCBs) in the main model but was greater than 95% for large risk differences between atosiban and CCBs (ie, RR values ≤0.53).

## Conclusions

In this nationwide cohort study of preterm newborns, in utero tocolytic exposure was not associated with increased or decreased risk of neurodevelopmental disabilities during early childhood (ie, by age 5.5 years) and there was no evidence of differences in neurodevelopmental disabilities by type of tocolytic treatment. Further research is needed to confirm these findings given that we cannot exclude a beneficial or detrimental association of small magnitude for tocolysis or according to tocolytic treatment type.

## Notes

Supplement 1. **eTable.** Comparison of Characteristics Between Mothers of Children Lost to Follow-Up at 5.5 Years and Those Participating at the Assessment at 5.5 Years **eFigure 1.** Balance Assessment of Maternal Covariates Before and After Matching for the First Analysis Comparing Groups of Infants Exposed to Tocolysis or Not **eFigure 2.** Balance Assessment of Maternal Covariates Before and After Matching for the Second Analysis Comparing Groups of Infants Exposed to Atosiban or Calcium Channel Blockers

Supplement 2.
Data Sharing Statement
