# Supplementary material for: Tocolysis and Neurodevelopment of Children Born Very Preterm
Source: JAMA Netw Open. 2024 Oct 31;7(10):e2442602. doi: 10.1001/jamanetworkopen.2024.42602 (PMC11528334; doi:10.1001/jamanetworkopen.2024.42602)
Supplement: Supplement 1. — eTable. Comparison of Characteristics Between Mothers of Children Lost to Follow-Up at 5.5 Years and Those Participating at the Assessment at 5.5 Years eFigure 1. Balance Assessment of Maternal Covariates Before and After Matching for the First Analysis Comparing Groups of Infants Exposed to Tocolysis or Not eFigure 2. Balance Assessment of Maternal Covariates Before and After Matching for the Second Analysis Comparing Groups of Infants Exposed to Atosiban or Calcium Channel Blockers [file jamanetwopen-e2442602-s001.pdf]

Supplemental Online Content

Plouchart T, Sabatier T, Muller J-B, et al. Tocolysis and neurodevelopment of children born very preterm. *JAMA Netw Open*. 2024;7(10):e2442602.
doi:10.1001/jamanetworkopen.2024.42602

eTable. Comparison of Characteristics Between Mothers of Children Lost to Follow-Up at 5.5 Years and Those Participating at the Assessment at 5.5 Years

eFigure 1. Balance Assessment of Maternal Covariates Before and After Matching for the First Analysis Comparing Groups of Infants Exposed to Tocolysis or Not

eFigure 2. Balance Assessment of Maternal Covariates Before and After Matching for the Second Analysis Comparing Groups of Infants Exposed to Atosiban or Calcium Channel Blockers

This supplemental material has been provided by the authors to give readers additional information about their work.

© 2024 Plouchart T et al. *JAMA Network Open.*

eTable 1 : Comparison of characteristics between mothers of children lost to follow-up at 5.5 years and those participating at the assessment at 5.5 years (n = 1 055)

Characteristics	No. of events/No. in group (%) [a] Participating at 5.5 years (n= 641)	Lost to follow-up at 5.5 years (n=414)	Chi-square P-value[b]
**Maternal characteristics**			
Age (years) (n=1 055)			
<25	99 (15.3)	102 (24.6)	<.001
25-34	434 (67.7)	240 (58. 0)	
≥35	108 (17.0)	72 (17. 4)	
Mother's country of origin (n=1 048)			
France or Europe	544 (85. 0)	317 (78.3)	
North African countries	42 (6.6)	35 (8.7)	.01
Other African countries	26 (3.9)	35 (8.2)	
Other	29 (4.5)	20 (4.8)	
Level of education (n=997)			
Less than high school	153 (25.0)	156 (41.0)	<.001
High school, 1-2 years of graduate studies	273 (44.1)	157 (41.2)	
3 years of graduate studies or higher	190 (30.9)	68 (17.8)	
Parents' socioeconomic position (n=1 009)			
Professional	173 (28.0)	68 (17.2)	
Intermediate	134 (21.6)	65 (17. 0)	
Administrative, public service	169 (27.8)	117 (29.9)	<.001
Service worker	64 (9.8)	62 (15.9)	
Manual worker	69 (11.1)	51 (13.1)	
Unemployed	10 (1.7)	27 (6.9)	
Smoking during pregnancy (n=1 021)	117 (18.9)	121 (30.6)	<.001
**Obstetrical characteristics and management**			
Twin gestation (n=1 055)	191 (29.9)	115 (27.6)	.43
Infertility treatment (n=1 016)	110 (17.5)	49 (12.1)	.02
Antenatal corticosteroids (n=1 025)	373 (60.4)	234 (58.1)	.46
In utero transfer (n=1 047)	282 (44.1)	181 (44.5)	.90
Antenatal magnesium sulfate (n= 1 041)	29 (4.7)	16 (3.8)	.50
Term of pregnancy at mother's admission (weeks) (n=1 050)			
<27	211 (29.2)	104 (21.8)	.03
27-29	221 (36.6)	162 (41.3)	
30-31	207 (34.2)	145 (36.9)	
Gestational age at birth, weeks (n=*1 055*)			
24-27	194 (26.3)	106 (22.1)	.10
28-31	447 (73.7)	308 (77.9)	
Tocolysis (n= 1055)	476 (74.1)	300 (72.5)	.57
**Maternity unit characteristics**			
Birth in a tertiary care center (n=1 055)	513 (80.0)	323 (78.0)	.44

[a] Denominators vary according to number of missing data for each variable. Percentages are weighted to take into account the varying survey durations among gestational age groups. [b] With Rao & Scott adjustment

© 2024 Plouchart T et al. *JAMA Network Open.*

eFigure 1. Balance Assessment of Maternal Covariates Before and After Matching for the first analysis comparing groups of infants exposed to tocolysis or not.

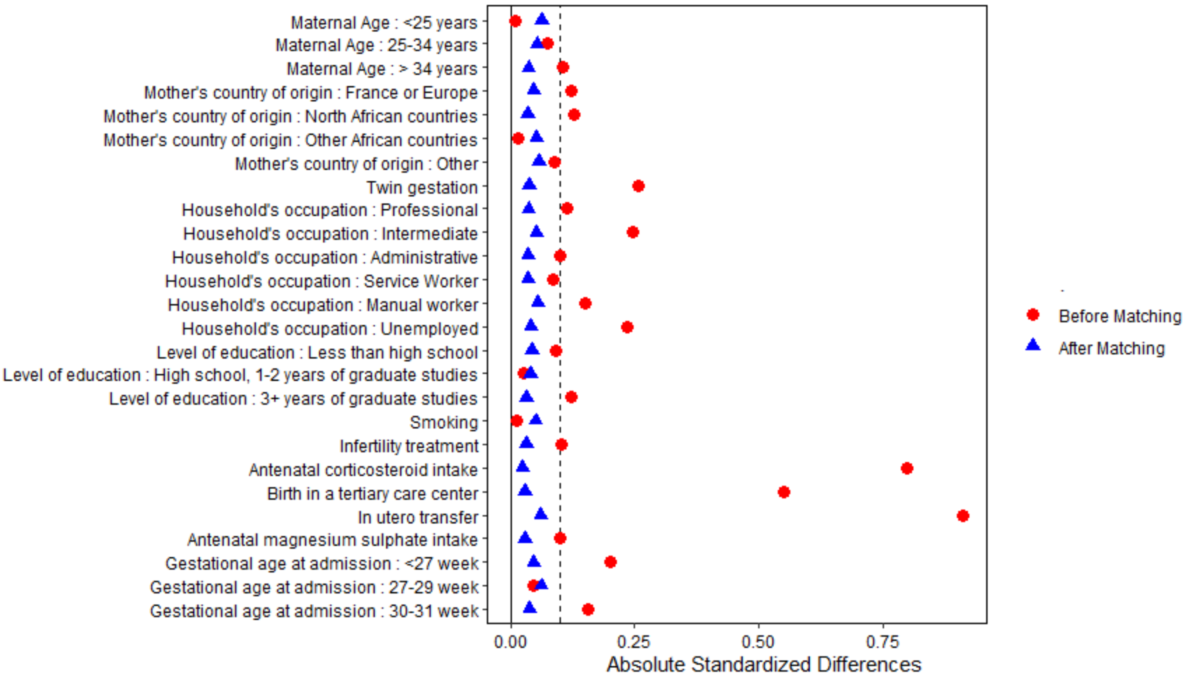

© 2024 Plouchart T et al. *JAMA Network Open.*

eFigure 2. Balance Assessment of Maternal Covariates Before and After Matching for the second analysis comparing groups of infants exposed to atosiban or Calcium channel blockers (CCBs)

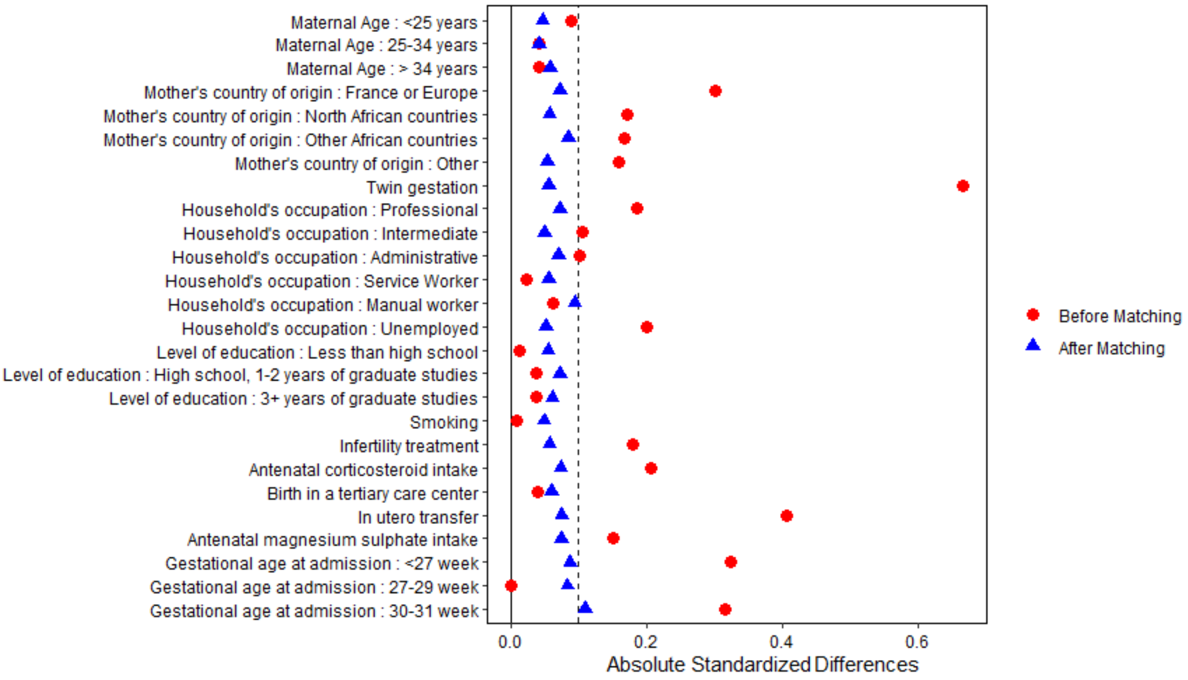

© 2024 Plouchart T et al. *JAMA Network Open.*
