# Supplementary material for: Tocolysis and Neurodevelopment of Children Born Very Preterm
Source: JAMA Netw Open. 2024 Oct 31;7(10):e2442602. doi: 10.1001/jamanetworkopen.2024.42602 (PMC11528334; doi:10.1001/jamanetworkopen.2024.42602)
Supplement: Supplement 2. — Data Sharing Statement [file jamanetwopen-e2442602-s002.pdf]

Data Sharing Statement

Plouchart. Tocolysis and Neurodevelopment of Children Born Very Preterm. *JAMA Netw Open*. Published online October 31, 2024. doi:10.1001/jamanetworkopen.2024.42602

Data
Data available: No

Additional Information
Explanation for why data not available: Research participants were guaranteed that the raw data they provided will remain confidential. To request access to the data, please send an email to Prof. Stephane Marret
